# Size-resolved simulation of particulate matters and CO_2_ concentration in passenger vehicle cabins

**DOI:** 10.1007/s11356-022-19078-1

**Published:** 2022-02-10

**Authors:** Dixin Wei, Filip Nielsen, Lars Ekberg, Jan-Olof Dalenbäck

**Affiliations:** 1 Volvo Car Corporation, Gothenburg, Sweden; 2 Division of Building Services Engineering, Department of Architecture and Civil Engineering, Chalmers University of Technology, Gothenburg, Sweden

**Keywords:** Particles, Filter, Modelling, PM_2.5_, UFP, Recirculation, Infiltration, Deposition

## Abstract

The main aim of this study is to develop a mathematical size-dependent vehicle cabin model for particulate matter concentration including PM_2.5_ (particles of aerodynamic diameter less than 2.5 μm) and UFPs (ultrafine particles of aerodynamic diameter less than 100 nm), as well as CO_2_ concentration. The ventilation airflow rate and cabin volume parameters are defined from a previously developed vehicle model for climate system design. The model simulates different filter statuses, application of pre-ionization, different airflow rates and recirculation degrees. Both particle mass and count concentration within 10–2530 nm are simulated. Parameters in the model are defined from either available component test data (for example filter efficiencies) or assumptions from corresponding studies (for example particle infiltration and deposition rates). To validate the model, road measurements of particle and CO_2_ concentrations outside two vehicles were used as model inputs. The simulated inside PM_2.5_, UFP and CO_2_ concentration were compared with the inside measurements. Generally, the simulation agrees well with measured data (Person’s *r* 0.89–0.92), and the simulation of aged filter with ionization is showing higher deviation than others. The simulation using medium airflows agrees better than the simulation using other airflows, both lower and higher. The reason for this may be that the filter efficiency data used in the model were obtained at airflows close to the medium airflow. When all size bins are compared, the sizes of 100–300 nm were slightly overestimated. The results indicated that among others, expanded filter efficiency data as a function of filter ageing and airflow rate would possibly enhance the simulation accuracy. An initial application sample study on recirculation degrees presents the model’s possible application in developing advanced climate control strategies.

**Supplementary Information:**

The online version contains supplementary material available at 10.1007/s11356-022-19078-1.

## Introduction

### Background

During the past decades, we have seen rising air quality problems, especially an increased number of airborne particulate matter. High particle concentrations have been found to influence human health. Smaller particles like PM_2.5_ (particles of aerodynamic diameter less than 2.5 μm) and UFPs (ultrafine particles, which have aerodynamic diameter less than 100 nm) have attracted focus due to their easier access into the human respiration system, and thus higher risks of lung and cardiovascular diseases (Mitsakou et al. 2007).

Considering the elevated particle concentrations on the road and increasing time spent in traffic, vehicle passengers are facing up even higher challenges of particle exposures (Zhu et al. 2007). Thus, there is a demand to get a better understanding of the air quality in vehicle cabins, and the influencing factors to support the development of corresponding protection systems, including filters and HVAC (heating, ventilation and air conditioning) system design.

There has been ongoing research on indoor air quality in buildings and different workplaces since decades ago, while the research about in-vehicle cabin air quality has developed during more recent years. Previous studies have reported about field measurements of in-cabin air quality in the form of particle concentrations, as well as the relations between inside and outside particle concentrations (Xu et al. 2018). There is also research based on modelling of the particle concentration, in turn based on field, as well as lab, measurements.

Complete vehicle measurements are relatively straightforward to perform, but they are expensive in terms of time and human resources. Measurements also include some uncontrollable variables, for example the ambient conditions, and measurement uncertainties. Alternatively, a simulation model could be implemented to mitigate the physical limitations of vehicle measurements, and moreover, to investigate scopes that cannot be realized in vehicle testing. A simulation model has, however, to be validated with complete vehicle measurements and laboratory measurements, to give trustworthy results.

Previous studies have modelled the in-cabin particle concentration and their influential factors, such as driving speeds, outdoor particles, ventilation airflows and infiltration (Xu and Zhu 2009; Gong et al. 2009; Joodatnia et al. 2013; Lee et al. 2015a; Ding et al. 2016). However, there appears to be a lack of studies which include the different filter statuses, pre-ionization, size-resolved filtration and air recirculation degrees, as well as a connection between air quality and climate energy consumption modelling.

### Aim of the study

The aim of the current study is to develop and evaluate a model of cabin PM_2.5_, UFP and CO_2_ concentration, to study the influence of filter performance and recirculation on cabin air quality and energy use. The model is based on a previous model of the vehicle climate system energy consumption (Nielsen et al. 2015). The previous model, developed in the software GT-SUITE, is complemented with a mass balance model where particle sizes, filter status, ventilation airflow and air recirculation degrees are considered. The influences of deposition and infiltration are also included. The cabin CO_2_ concentration, an established indicator for air quality, is also modelled, considering that air recirculation might cause accumulated CO_2_ (Kilic and Akyol 2012; Luangprasert et al. 2017). The model is validated against previous vehicle measurements performed under various conditions (Wei et al. 2020).

Field measurements, as well as model simulations, suggest that improved filtration is the most important action to improve the air quality in passenger car cabins. The main requirements of a model, besides the possibility to evaluate different filtering arrangements, is also the possibility to evaluate different ventilation strategies to improve the air quality, e.g. by recirculation, and reduce the use of energy for air conditioning.

## Methods

The research comprises literature studies, model development and validation, and an initial sample demonstration of the model capabilities. First, the basic model concept is explained. Second, the details of model parameter definition are explained. Third, the model validation process using previous road-testing data is explained.

### Model development

#### ***Background: climate system model***

The model development is based on an extension of a previously developed vehicle climate system model (Nielsen et al. 2015). In that model, the vehicle climate components and control strategies were simulated in detail. The software GT-SUITE, which solves the Navier–Stokes equation in one dimension, was used to simulate the climate systems. The climate system model focused on the energy consumption of the climate system, and pure air without pollutants was assumed as incoming air. Filtration of particles was neglected while the pressure drop at the filter was considered. For more details, please see the ‘Methods’ chapter of the published paper. In that model, the relevant sub-modules for this study are passenger compartment and air handling modules, i.e. the airside model. The next sections will explain how the particle and CO_2_ model is developed based on the climate system model.

The vehicles being modelled in this study are the same as in a previous vehicle measurement study (Wei et al. 2020), i.e. a Volvo XC90 (model year 2018) with an estimated cabin volume of 4.1 m^3^, and a Volvo S90 (model year 2018) with an estimated cabin volume of 2.9 m^3^. The two test vehicles share the same HVAC system design and climate control systems.

#### ***Airside model with particles and CO***_***2***_

**Fig. 1:**
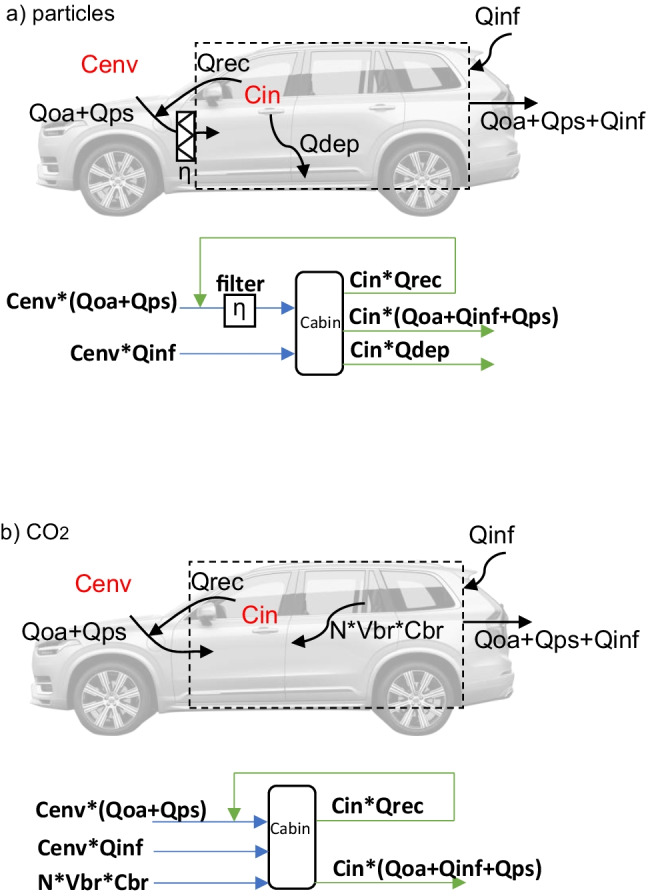
Illustration of **a** particle and **b** CO_2_ transport in vehicle cabins and the corresponding simplified flowcharts where the losses and gains of the cabin are marked green and blue

Figure 1 illustrates the basic particle/CO_2_ transport in vehicle cabins. For particles, the outside ventilation airflow (*Qoa*) with a particle concentration of *Cenv* enters the vehicle cabin through the HVAC system, and mixes with the recirculated airflow (*Qrec*) before passing the filter. Besides, the passive ventilation airflow (*Qps*) refers to the air entering the HVAC system, which is not induced by the operation of the fan, but because of for example the vehicle’s speed or wind speed. This flow is accounted for in the total ventilation flow in the studied vehicles and it also passes the filter (Ott et al. 2008; Lee et al. 2015a). Thus, it is not considered infiltration. The filter removes particles by an efficiency value of *η* (within 0 to 1), which is size dependent. The recirculation degree (%) defines the ratio of *Qrec* to *Qrec* + *Qoa*. The infiltration airflow (*Qinf*) here refers to the uncontrolled air leakage through cracks and leaks on the vehicle envelope, for instance cracks between the frame and doors (Xu et al. 2010). Particle deposition flow on the interior surfaces like seats and carpets is described as *Qdep*. *Cin* and *Cenv* are inside and outside particle concentrations.

For the CO_2_ air transport, the transport is similar except for that CO_2_ is not removed by the HVAC filter, and not deposited on the surfaces. Besides, the internal source from human breath is added, where *N* is the number of passengers, *Vbr* is minute ventilation in litres per minute and *Cbr* is the carbon dioxide concentration contained in the exhaled air. *Cin* and *Cenv* are inside and outside concentrations of CO_2_.

1 $$\begin{array}{c}\frac{dCin}{dt} \times Vcabin=(\left(Qoa+Qps) \times \left(1-\eta \right)+Qinf \times \alpha \right) \times Cenv-\\ \left(Qoa+Qps+Qinf+Qdep+Qrecx\eta \right) \times Cin\end{array}$$

2 $$\begin{array}{c}\frac{dCin}{dt} \times Vcabin=\left(Qoa+Qps+Qinf\right) \times Cenv+N \times Vbr \times Cbr-\left(Qoa+Qps+Qinf\right) \times Cin\end{array}$$

Based on the transport mechanisms in Fig. 1, the corresponding mass balance equations for the vehicle cabin are given in Eqs. (1) and (2), where the in-cabin concentration (*Cin*) of particles and CO_2_ are estimated correspondingly. The estimation uses inputs from parameters including outdoor particles/CO_2_ levels, vehicle speed, ventilation airflow (climate settings), filter status, ionization status, passenger numbers etc. The penetration loss coefficient *α* is accounting for the loss of particles at cracks through which the infiltration flow passes, and an experienced value of 0.6 is utilized (Xu et al. 2010) in Eqs. (1) and (2). *Vcabin* is the vehicle cabin volume (m^3^).

It should be noted that the particles considered in this study vary between 10 nm and 2.5 μm, i.e. PM_2.5_ except for particles less than 10 nm. The lack of the smallest particles is due to the instrument detection limit in road measurements. The size range is divided into 25 size channels in accordance with the instruments. *Cenv* and *Cin* are concentrations of particles in certain size channels, and *η* is the corresponding filtration efficiency of particles in that size channel. The balance equation was originally deployed for particle count concentration, considering that the definition of filtration efficiency is based on particle counts (number of particles per unit volume). Under this condition, *Cenv* and *Cin* represent count concentration per size channel (N/cm^3^), while the equation can also be used for mass concentration, since it is size dependent, i.e. assuming particles in the same size channel have the same average aerodynamic diameter and density. Thus, the particle mass concentration per size channel (μg/m^3^) was simulated with the same equation.

To solve Eqs. (1) and (2), the parameters require definitions based on the application conditions. In this study, the parameters are either defined from available test data (*η*, *Cenv* for particles, *N*), defined from a previously developed model (*Qoa*, *Qrec*, *Vcabin*) or based on experience from relevant studies (*Qdep*, *Qinf*, *Nbr*, *Cbr*, *Qps*, *α*, *Cenv* for CO_2_). Then, the steady-state solution of *Cin* under given conditions can be calculated. The details about all parameter definitions in Eqs. (1) and (2) will be presented now in each corresponding section.

##### Ventilation airflow and cabin volume

For the two modelled vehicles in this study, the previous climate system model calculates steady-state results of airflow rate simulation (*Qoa*, *Qrec*) based on relevant model inputs, including HVAC fan speed, vehicle speed, recirculation degrees, HVAC flap positions, ambient temperatures etc. These values were obtained from the validation measurement data, through either the vehicle’s own logs or the instrument. The cabin volume *Vcabin* was also estimated from the same model.

The passive ventilation airflow *Qps* entering the cabin has been found linearly related to the vehicle driving speed *vspeed* (Ott et al. 2008). Linear regression of the measured passive ventilation data has reported an experience coefficient of 0.21 m^−1^(Lee et al. 2015b). Thus, *Qps* is calculated as in Eq. (3) in this study.

3 $$\begin{array}{c}Qps=0.21 \times vspeed \times Vcabin\end{array}$$

##### Incoming particles and filtration of particles

To investigate the air quality features in this study, particles were added into the incoming air in the previous climate model in the software GT-SUITE, to simulate the particles from the environment. Besides, the filtration of particles was implemented in the filter component, where *η* is defined based on available component data. Details are given in the next paragraphs.

The atmospheric particles consist of varying compositions depending on the particle sources, type, locations etc. To simulate the particles species in the software GT-SUITE, a FluidGas template is used to simulate particles as Tracer Gas, where the concentration of particles could be defined regardless of chemical compositions (Gamma Technologies llc 2019). An injection template is used at the HVAC inlet position to mix incoming air with particles, where the outside particle concentration *Cenv* (μg/m^3^), outside airflow rate *Qoa* (m^3^/s) and passive ventilation airflow *Qps* (m^3^/s) are defining the injection airflow rate (μg/s).

On the other hand, the filtration process of particles is simulated with an ejection template at the filter component, where the ejection rate of particles is defined with the size-dependent filter efficiency *η*. This study simulated the same filters used in the validation measurements (Wei et al. 2020), which are a newly manufactured filter and a 500-h-aged (end of service interval) filter of the same type. For the new filter status, the efficiency values were applied from several available supplier component tests. For the 500-h-aged status, several component test data are also available for the same filter model type, although less than the new filter status. Similarly, the filter efficiency data with pre-ionization are based on a restricted number of test data, which means the efficiency for an aged filter with ionization was partially estimated based on the ionization improvement on the new filters. The tests were mainly performed under an airflow of 288 m^3^/h (80 L/s), and thus, the influence of airflow on filter efficiency is not considered in the initial model.

During the simulation, given the filter status and ionization status, the corresponding upper and lower limits of all the available efficiencies are used for *η*, which are given in Table 1. With above application in the model, the items (*Qoa* + *Qps*) ∗ (1 − *η*) are exported from GT-SUITE steady-state simulation results as the input to Eqs. (1) and (2). Later the simulated *Cin* with two sets of *η* are averaged as the average simulated in-cabin particle concentration.

**Table 1:** Size-dependent filter efficiency *η* applied in the simulation. The values are given for different filter status (new and aged) as well as ionization status (on and off). The upper and lower limits correspond to the range of efficiencies obtained in different component tests for the same condition

Conditions		Particle size channels (nm)																								
**Filter status**	**Pre-ionization status**	**10**	**14**	**19**	**27**	**37**	**52**	**72**	**100**	**139**	**193**	**253**	**298**	**352**	**414**	**488**	**576**	**679**	**800**	**943**	**1112**	**1310**	**1545**	**1821**	**2146**	**2530**
**Upper limit**																										
New	Off	0.79	0.79	0.79	0.79	0.79	0.79	0.78	0.76	0.76	0.71	0.68	0.68	0.68	0.98	0.99	0.99	0.99	0.99	0.99	0.99	0.99	0.99	0.99	0.99	0.99
500-h-aged	Off	0.56	0.56	0.56	0.56	0.56	0.56	0.49	0.42	0.42	0.33	0.33	0.29	0.29	0.66	0.67	0.67	0.67	0.67	0.67	0.67	0.67	0.67	0.67	0.67	0.67
New	On	0.88	0.88	0.88	0.88	0.88	0.88	0.88	0.88	0.90	0.91	0.92	0.92	0.93	0.99	0.99	0.99	0.99	0.99	0.99	0.99	0.99	0.99	0.99	0.99	0.99
500-h-aged	On	0.65	0.65	0.65	0.65	0.65	0.65	0.58	0.51	0.51	0.42	0.42	0.38	0.38	0.75	0.76	0.76	0.76	0.76	0.76	0.76	0.76	0.76	0.76	0.76	0.76
**Lower limit**																										
New	Off	0.65	0.65	0.65	0.65	0.65	0.65	0.65	0.65	0.64	0.64	0.61	0.63	0.69	0.77	0.77	0.77	0.82	0.84	0.84	0.89	0.89	0.92	0.92	0.95	0.95
500-h-aged	Off	0.25	0.25	0.25	0.25	0.25	0.25	0.25	0.25	0.25	0.25	0.25	0.25	0.25	0.37	0.37	0.37	0.42	0.44	0.44	0.49	0.49	0.52	0.52	0.55	0.55
New	On	0.87	0.87	0.87	0.87	0.87	0.87	0.87	0.87	0.87	0.87	0.87	0.87	0.87	0.87	0.94	0.94	0.95	0.96	0.96	0.94	0.94	0.98	0.98	0.95	0.95
500-h-aged	On	0.34	0.34	0.34	0.34	0.34	0.34	0.34	0.34	0.34	0.34	0.34	0.34	0.34	0.46	0.46	0.46	0.51	0.53	0.53	0.58	0.58	0.61	0.61	0.64	0.64

##### Deposition and infiltration

In this section, the definitions of particle deposition flow (*Qdep*) and infiltration airflow (*Qinf*) in Eqs. (1) and (2) are explained. The *Qdep* in the vehicle cabin could be modelled using the deposition rate *β* (h^−1^) as in Eq. (4). The deposition rate value has been reported to be 0.6–12 h^−1^ for PM_2.5_ by Harik et al. (2017) and 3.2–11.8 h^−1^ on average for UFPs by Gong et al. (2009). The variation is due to vehicle type, airflow rate and particle size. Based on the studied vehicles and airflows in this study, the size-dependent depositions rates deployed for particles between 10 nm to 2.5 μm are summarized in Appendix Table A1.

4 $$\begin{array}{c}Qdep= Vcabin \times \beta \end{array}$$

Lee et al. (2015b) have performed studies on the infiltration airflow through both experimental measurements and modelling analysis. They concluded methods to model infiltration flow as in Eqs. (5) to (8). Equation (5) explains the pressure difference caused by mechanical ventilation of outside air (*Qoa*) and passive ventilation airflow (*Qps*). The leakage parameters *kf* and *n* depend on vehicle types and was measured for 10 vehicles from cabin pressurization tests (Lee et al. 2015a). While driving, the differential pressure on outer surface of the vehicle caused by aerodynamic changes (*dPaero*) could be derived as Eq. (6) from vehicle speed (*vspeed*) and vehicle characteristic parameters *a*, *b* and *kp*. When the pressure at outer surface is higher than the cabin pressure ($\Delta Pinf>0)$, infiltration could occur due to the pressure difference. This infiltration flow *Qinf* is calculated as Eq. (8), where *Frev* is the reverse leakage flow correction factor which considers the difference between infiltration flow and exfiltration flow (Fletcher and Saunders 1994). In this study, the vehicle-related parameter values (*kf*, *n*, *a*, *b*, *kp*, *Frev*) adopted from previous studies are presented and explained in Appendix Table A2.

5 $$dPmech={e}^{\frac{1}{n}\mathrm{ln}\left(\frac{Qoa+Qps}{kf}\right)}$$

6 $$dPaero=kp \times a\bullet {e}^{b\bullet vspeed}$$

7 $$\Delta Pinf=dPaero-dPmech$$

8 $$Qinf=Frev\bullet kf\bullet {\Delta Pinf}^{n}$$

##### Assumptions on respiration losses/gains

The passengers’ respiration losses/gains of particles in the cabin are considered negligible compared with losses from filtration and deposition. This assumption is supported by Xu and Zhu (2009), that respiration airflow is nearly zero under driving conditions, and even under extreme idling conditions, the deposition losses are 40–210 times higher than respiration losses. Besides, no phase change of particles is included in the model. The model assumes air in the cabin is well-mixed, i.e. the particle concentration is the same in different positions, which was reported in previous four-point particle measurements in vehicles (Joodatnia et al. 2013).

#### ***CO***_***2***_*** parameters***

The outside and recirculation airflow rate parameters (*Qoa*, *Qps*, *Qrec*) used in the CO_2_ model in Eq. (2) are the same as the particle models in Eq. (1), while the unique CO_2_ parameters that require definition are *Cenv*, *Cbr*, *Vbr* and *N*. *Cenv* represents the outside CO_2_ concentration (ppm). The internal generation of CO_2_ from passenger respiration exhalation is another source. It is simulated based on the number of passengers (*N*), the average CO_2_ concentration contained in exhaled air (*Cbr*) and the average exhaled air volume (*Vbr*). The definitions of these parameters are explained further in following paragraphs.

##### Atmospheric CO_2_ concentration

The outside CO_2_ concentration *Cenv* can be determined either from measurement data or from estimations based on the vehicles’ incoming air conditions. As will be explained in next section, the validation process of this study utilized road measurement data which contains in-cabin CO_2_ concentrations throughout the whole campaign, while not the simultaneous outside CO_2_ concentration. Thus, an estimation of the outside CO_2_ (*Cenv*) is used in the validation.

The vehicle measurement was performed in a road tunnel in Gothenburg, Sweden, in 2018. The tunnel environment was identified to have elevated CO_2_ concentrations compared to the open-road environment, due to weakened diffusion of vehicular emitted pollutants as well as higher traffic density (De Fré et al. 1994; Cong et al. 2017; Wei and Wang 2020). Considering the tunnel length, sampling location, traffic density and vehicle compositions, the corresponding data from Ho et al. (2009) and Zhang et al. (2015) were considered comparable, which were 710 ppm and 722 ppm respectively. The average of two, 716 ppm, was used for the parameter *Cenv* in model validation.

##### Respiration-exhaled CO_2_

As described in ‘Model validation’, the respiration-exhaled CO_2_ source is added in Eq. (2) as *N* ∗ *Vbr* ∗ *Cbr*. *Vbr* represents minute ventilation (or respiratory minute volume) in litres per minute, which is the gas volume exhaled from a person’s lung per minute. It varies with physical activity levels and personal characteristics. Minute ventilations under normal sitting conditions vary between 5 and 8 L/min (Levitan 2015). An average *Vbr* of 6.5 L/min is used in this study since passengers sitting in a standstill car were almost at rest. *Cbr* is the carbon dioxide concentration contained in the exhaled air (ppm). According to a previous study on carbon dioxide exposure (Scott et al. 2009), *Cbr* is set to 40,000 ppm. *N* is the number of passengers, which is defined from measurement logs and is either 2 or 3 people in the vehicle.

### Model validation

The model validation uses results from previous vehicle measurement on roads. The measurements were performed in two locations, Sweden and Northern China, under similar testing setups: varied filter status, airflow rates, recirculation degrees, utilization of pre-ionization etc. After the parameters were varied to each test case, the steady-state values were obtained. The measured values are both in-cabin and outside particle counts and mass concentrations for 41 size channels from 10 nm to 35 μm, as well as PM_2.5_ and UFP concentrations. The simultaneous in-cabin CO_2_ concentration was logged as well in Sweden for all the test cases. More detailed descriptions can be found in the methods section of the previous paper by Wei et al. (2020).

**Fig. 2:**
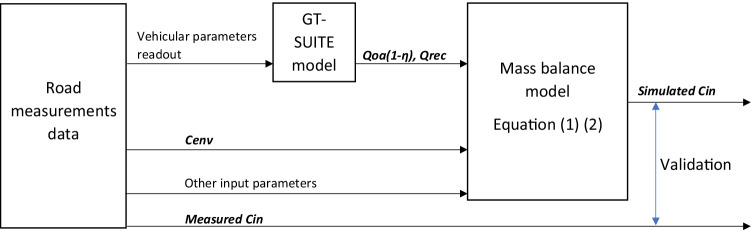
Model validation process illustration

The validation process is illustrated in Fig. 2. To validate the road measurements, the actual HVAC fan speed, flap positions, vehicle speeds, ventilation setup of recirculation degrees and air distribution were read from the test data and input into the air quality model in GT-SUITE, to simulate the measurement conditions. Based on the filter used, the corresponding efficiencies as from Table 1 are used in the model as well. With these setups, the HVAC outside airflow rates (*Qoa*), recirculation airflow rates (*Qrec*) and the item *Qoa* ∗ (1 − *η*) are obtained from steady-state simulation results. Besides, the measured outside particle or CO_2_ concentration were read as *Cenv*. Together with other input parameters as explained in previous sections (*Qdep*, *Qinf*, *Nbr*, *Cbr*, *α*, *N*), the steady-state solution of *Cin*, i.e. the simulated in-cabin particle concentration for each particle size, or CO_2_ level could be compared with the actual measurements. When the particle concentrations within certain sizes are summarized, the simulated PM_2.5_ (μg/m^3^) and UFP counts (N/cm^3^) are obtained and can be compared with the real road measurement.

## Results

### *Overall validation of PM*_*2.5*_*, UFP and CO*_*2*_

**Fig. 3:**
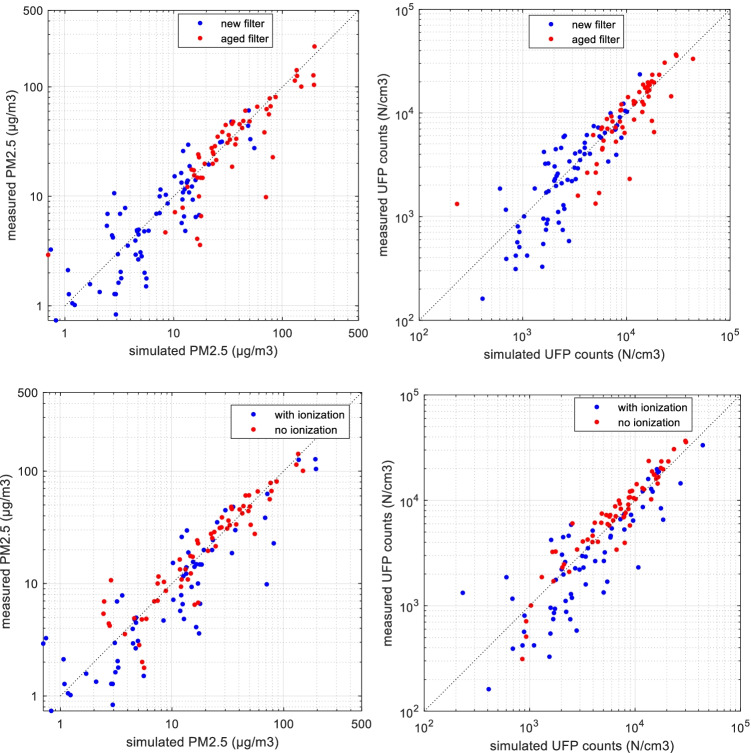
Comparison of simulated and measured in-cabin PM_2.5_ values and UFP counts. Data include all test cases (128 samples), including both new and aged filter statuses, ionization and no ionization

The simulation data are compared with steady-state road measurements, both regarding the total particle concentration (PM_2.5_, UFP), the particle concentration per size channel and the CO_2_ concentration. As mentioned previously, a filter efficiency range is adopted for the parameter *η*, which results in a model concentration range. As in Fig. 3, the measured and simulated (average of model concentration range) inside PM_2.5_ values (μg/m^3^) of all steady-state test cases are presented, and both new and aged filter statuses are included, as well as ionization and no-ionization groups are marked. The UFP counts (N/cm^3^) are also compared.

The two particle sizes show similar trends, that most of the simulation values correlate with measurements well, with some separate cases showing deviation. This similar trend is expected since the particles smaller than 100 nm account for a large part of the total measured counts, due to sources from road vehicular emissions (Qi et al. 2008). For part of the tests in Sweden, when the outdoor air is relatively clean, and the new filter is installed, the inside PM_2.5_ is lower than 10 μg/m^3^. At this range, the simulation scatters relatively more due to low absolute particle levels. When comparing the filter statuses, the aged filter generally results in higher in-cabin particle levels due to deteriorated filtration, while the overall comparison between simulation and measurement is quite similar when the two locations are compared. The Pearson’s correlation coefficient (*r*) is calculated between simulation and measurement for different groups in Fig. 3 respectively. The new filter group has *r* of 0.87 compared with 0.90 in the aged filter group. The ionization group has *r* of 0.92, and the no-ionization group has 0.96 (all *p* < 0.05).

**Fig. 4:**
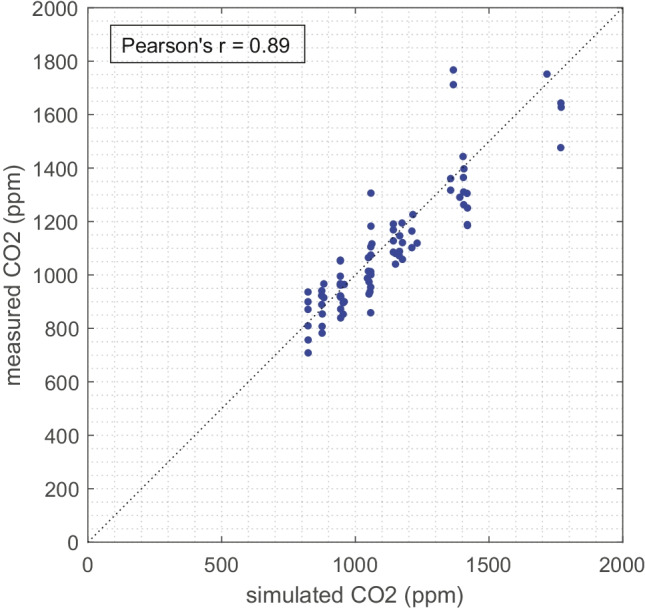
Comparison of simulated and measured steady-state in-cabin CO_2_ concentrations (ppm). Data include all test cases (81 samples)

Similarly, the comparison of CO_2_ concentration prediction and measurement is shown in Fig. 4. The prediction generally agrees well within the measurement range and the Pearson’s *r* is 0.89.

To further evaluate the model performance, several model performance factors as in Table 2 are calculated. The definition and explanations of these parameters are given in Eqs. (10)–(15) in Appendix B. These parameters are selected to reflect both mean bias and random scatter (Patryl and Galeriu 2011). Especially since the data of in-cabin particle concentration (PM_2.5_) exist in a wide range of magnitudes due to different measurement locations, the use of logarithmic forms (*MG* and *VG*) are considered appropriate (Hanna et al. 1993). When using these parameters, Joodatnia et al. (2013) proposed that a good atmospheric model prediction should meet the following criteria:

- The mean bias should be within 30% of the mean: 0.7 < *MG* < 1.3 and $\mid$ *FB*∣ < 0.3.
- Random scatter of predictions within a factor of 2 of the mean: *VG* < 1.6 and *NMSE* < 4.
- *FAC2* > 0.7.
- Pearson’s *r* > 0.7.

**Table 2:** Model performance evaluation factors and index to corresponding definitions

Factor	Name	Definition in Appendix B
*r*	Pearson correlation coefficient	Equation (10)
*FAC2*	The fraction of predictions within a factor of 2 of observations	Equation (11)
*FB*	Fractional bias	Equation (12)
*MG*	Geometric mean bias	Equation (13)
*NMSE*	Normalized mean square error	Equation (14)
*VG*	Geometric variance	Equation (15)

The calculated factors for this model are presented in Table 3. The predictions of particles and CO_2_ meet all the above criteria well. Generally, the prediction shows good performance. The CO_2_ model prediction shows slightly less deviation, possibly due to the model not containing variance from filtration as particle models.

**Table 3:** Statistical performance of modelled particle concentrations and CO_2_ concentrations versus corresponding measurements

Modelled concentration	Pearson’s *r*	*FAC2*	*MG*	*VG*	*FB*	*NMSE*
PM_2.5_ (μg/m^3^)	0.92	0.81	0.90	1.37	− 0.10	0.34
UFP counts (N/cm^3^)	0.91	0.81	0.90	1.35	0.00	0.17
CO_2_ (ppm)	0.89	1.00	0.98	1.01	− 0.02	0.01

### Validation of filter statuses, ventilation airflows and recirculation

As Fig. 3 provides an overall profile of the simulated and measured particle concentrations in the cabin, these results could be further investigated within different categories. For example different filter ages and ionization statuses were included, which have significant influence on the filtration performance, i.e. the filter efficiency (*η*). The simulation performance could thus be influenced by these parameters.

So, the results were classified into 4 categories, considering new and aged filters, as well as ionization on and off. It was noted that within each category, the outside particle concentrations (*Cenv*) were distributed in a wide range. To be able to compare, the indoor to outdoor ratio (*I*/*O* ratio) is considered, which is the inside concentration divided by outside concentration for PM_2.5_ or UFP counts.

**Fig. 5:**
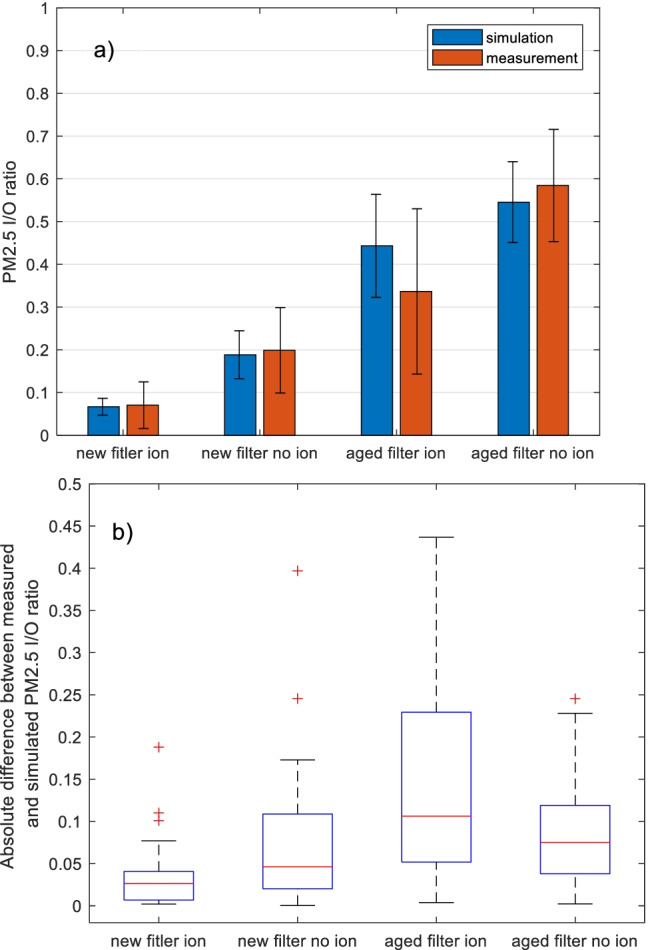
PM_2.5_ indoor to outdoor ratios (I/O ratios) are compared in different parameter groups: the filter statuses of new and aged, and the ionization status of on and off are combined: **a** average simulated and measured I/O ratios are compared; error bars present standard deviation. **b** The absolute differences between simulated and measured I/O ratios of each sample are summarized in box-whisker plots. Data include all test cases (128 samples)

As in Fig. 5a, the simulated and measured average PM_2.5_ *I*/*O* ratios of each category are compared. The simulations towards new filters give close average *I*/*O* ratios to the measurements, where the differences between averages are all within 5%. On the contrary, the aged filter ionization category showed the largest deviation of simulated *I*/*O* ratio (31%), and this group also shows larger variance (*Std* = 0.19) of the measured *I*/*O* ratio compared with others, which is possibly due to the particle accumulation not being even throughout the aged filter surface, and thus possibly more unstable performance. It could also be seen from the graph that the simulation tends to overestimate the *I*/*O* ratio for the aged filter ionization group, i.e. underestimate the filtration performance.

Furthermore, in graph b, the difference between the PM_2.5_ *I*/*O* ratio for each sample is calculated, and then summarized under the four categories using the box-and-whisker plots. So, each column shows the distribution of simulation and measurement deviation, in the sense of *I*/*O* ratio. It confirms the observation from graph a that the aged filter ionization group has a higher simulation deviation, and the *I*/*O* ratio difference also lies in a wider range.

Similarly, the results are also analysed for UFPs, and the trends are similar to PM_2.5_. They are given in Appendix C Fig. 13. A minor difference is that absolute *I*/*O* ratio differences in the aged filter ionization group are less scattered.

**Fig. 6:**
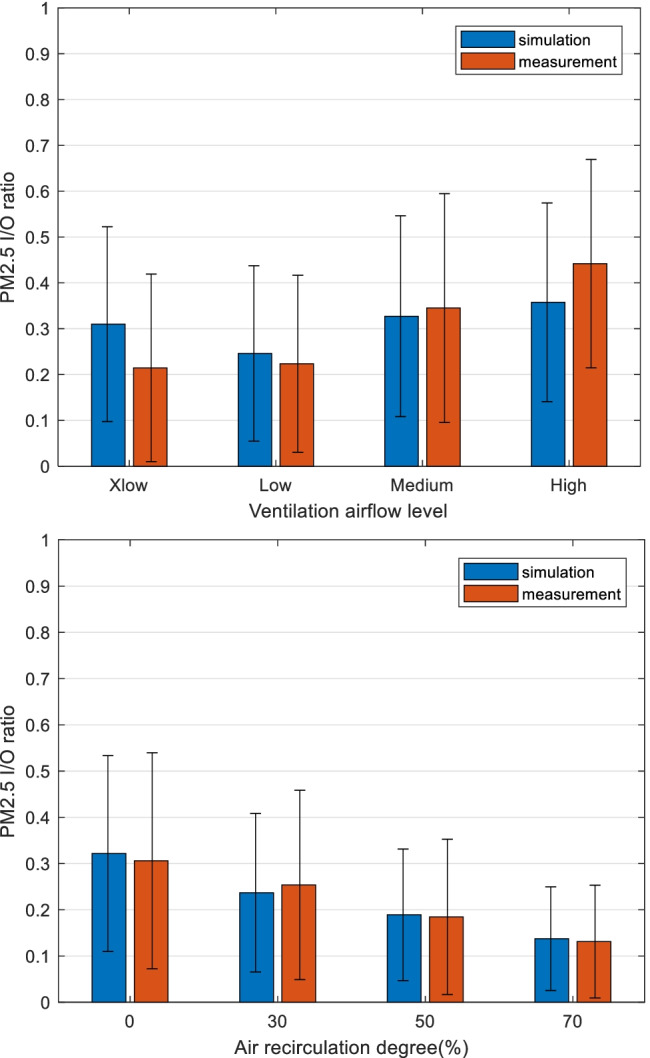
PM_2.5_ indoor to outdoor ratios (*I*/*O* ratios) are compared in different airflow levels and air recirculation degrees: average simulated and measured *I*/*O* ratios are presented and compared. Error bars present standard deviation. Data include all test cases (128 samples)

The performance of simulation under different ventilation airflow levels are compared in Fig. 6. The simulated and measured PM_2.5_ *I*/*O* ratios are compared under 4 different ventilation levels (Xlow, Low, Medium and High). The estimated airflow rates at these 4 levels are around 23, 40, 59 and 86 L/s respectively. It should be noted that the relatively large standard deviations are due to the variation of filter statuses, ionizations etc.

When comparing the simulation and measurement, the Medium airflow category is closer to reality, which could be related to the filter efficiency (*η*) estimation as shown in Table 1. These filter efficiency values are mainly from filter component tests under standardized airflow rates of 288 m^3^/h (80 L/s), which is between Medium and High levels in the simulated cars. Since the filter efficiency is influenced by the ventilation airflows in reality (Knibbs et al. 2010; Shi 2012), this estimation could cause the deviation for the other airflows when only efficiencies under one airflow are utilized. Furthermore, 4 paired samples *t*-tests between the simulated and measured PM_2.5_ *I*/*O* ratios in each airflow level are performed, at a significance level of 0.05. The corresponding *p* values are 0.00, 0.05, 0.56 and 0.01, which confirms that the Medium airflow category showed no statistically significant difference between simulation and measurement averages.

Moreover, the general trend is that higher ventilation airflow rates lead to higher *I*/*O* ratios, since a shorter residence time deteriorated the filtration capability (Qi et al. 2008). Another possible cause could be that the relative importance of the effect of deposition diminishes as the ventilation rate increases. But the Low ventilation category in Fig. 6 contains half of the samples with recirculation while the other three categories do not, which resulted in lower *I*/*O* ratios due to less outdoor particles.

Similarly, the 4 air recirculation degrees (%) are compared in Fig. 6, where higher recirculation degrees lead to lower measured *I*/*O* ratios. It is also seen that the average of simulation is close to average of measurement in all 4 levels and the recirculation estimation is not influencing the simulation performance to a high degree. This agrees with the simulation process since the recirculation is not influencing the estimated filter efficiency.

### Validation of particle size

**Fig. 7:**
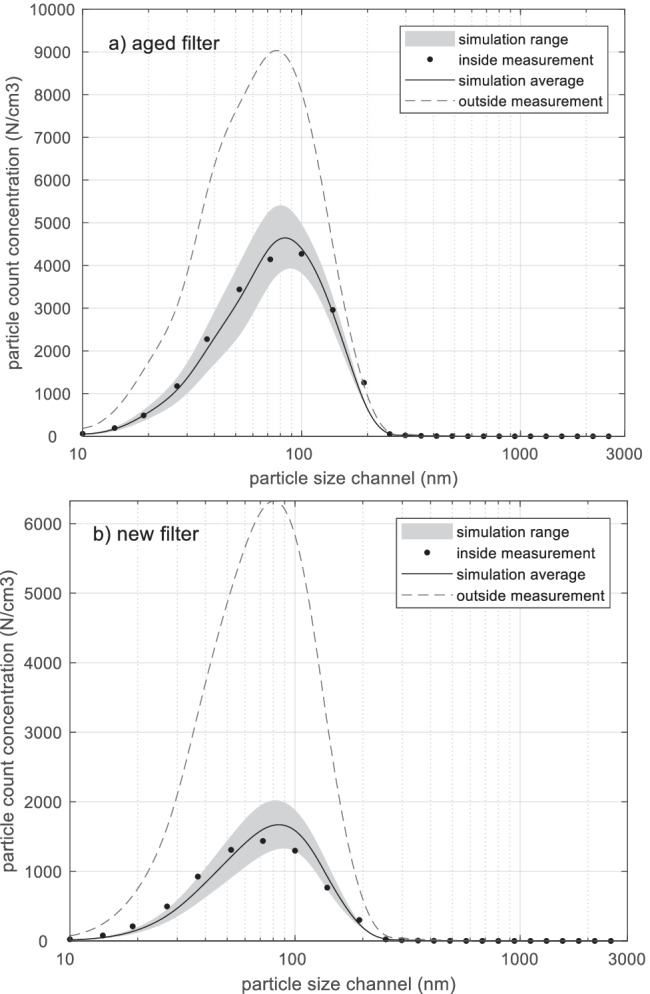
Two example cases are presented regarding measured particle count concentration (inside and outside) and simulated particle count concentration range/average per size channel. The two examples show mean two 5–10-min stabilization measurements under these corresponding settings: **a** aged filter, no ionization, Low airflow rate, Sweden; **b** new filter, no ionization, Middle airflow rate, Sweden

The simulation of particle concentration is also evaluated for each size channel, which includes 25 size channels from 10 nm to 2.5 μm. Firstly, two example test cases are chosen for visualization of comparison in Fig. 7. The measured inside particle count concentration (N/cm^3^) per size channel, the predictions and the corresponding outside measurement are shown. The grey area represents the model range due to variations in the filter efficiency parameter, and the model average is also given. The measurement value mostly lies in the prediction range, and the range around 100–200 nm is slightly overestimated in case b. It is obvious that the aged filter is less efficient at removing outside particles.

To further investigate the bias in each size channel, the fractional bias (*FB*) between average simulated and measured particle counts was calculated in different size channels for the new and the aged filter separately, and sizes larger than 352 nm are excluded since particle counts are nearly zero. The dimensionless *FB* is used since inside particle concentrations (*Cin*) alter widely within different sizes. *FB* reflects the mean bias between prediction and measurement, i.e. an evaluation of overestimation or underestimation, as in Eq. (9), where *P* is a predicted concentration and *O* is the corresponding observed concentration. Thus, a negative *FB* value shows an overprediction and reversely positive *FB* indicates an underprediction. *FB* that is equal to 0.67 is equivalent to underprediction by a factor of 2 (Patryl and Galeriu 2011).

9 $$\begin{array}{c}\frac{\overline{P} }{\overline{O} }=\frac{1-0.5FB}{1+0.5FB}\end{array}$$

**Fig. 8:**
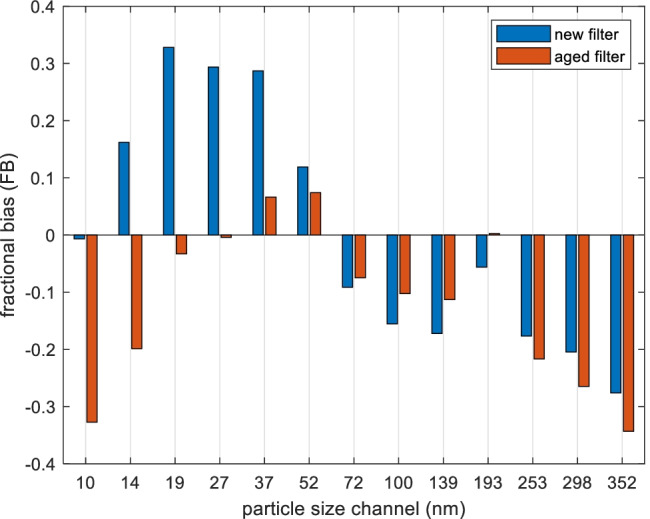
Fractional bias (*FB*) per size channel, categorized with filter type. Data include all test cases (128 samples)

The *FB* values of each size channel for the two filter types are shown in Fig. 8. The *FB* values are mostly within ± 0.3, which could be considered good. The 52–352-nm range normally contains the most particle counts, and the model prediction here mostly shows a slight overestimation of concentrations in both new and aged filter groups.

### Sensitivity analysis on parameters in the simulation

The parameters utilized in the simulation for estimation of filter efficiency, ventilation airflow, infiltration and deposition are investigated in this section, and the corresponding sensitivity analyses are presented. The UFP results are similar to that of PM_2.5_ and are thus not presented.

#### Filter efficiency

Filter efficiency (*η*) is a crucial factor in the filtration modelling (Xu et al. 2011; Shi 2012), especially within the size range where most particles are distributed. As shown in Fig. 7, the highest inside particle concentrations normally are found in a narrow size range around 100 nm. One reason for this is that the outdoor concentration is high in that size range. Another reason is that the most penetrating particle size range of the cabin air filter is within, or close to, that size range (Xu et al. 2013). On average, the particle count concentration (N/cm^3^) in the size range 52–352 nm comprises 68% of the total inside count concentration. The mass concentration (μg/m^3^) comprises 83% of the total inside mass concentration in our measurements.

**Fig. 9:**
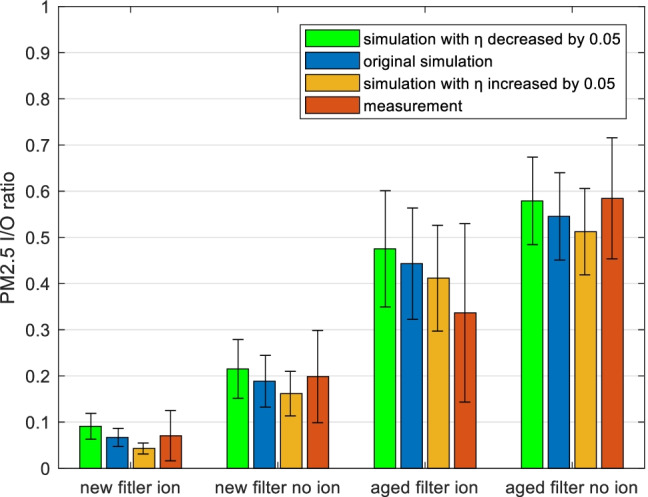
Comparison between simulated PM_2.5_ ratios when filter efficiency (*η*) is increased and decreased by an absolute value of 0.05 and measurement. The data are divided into 4 categories: the filter statuses new and aged, and the ionization statuses on and off are combined, which is the same as in Fig. 5. Error bars present standard deviation. Data include all test cases (128 samples)

Thus, to investigate the influence from filter estimation, the filter efficiency parameter (*η*) is altered ± 0.05 in the size range 52–352 nm. The changes of the simulation results are shown in Fig. 9.

Based on Fig. 5, the average simulated PM_2.5_ *I*/*O* ratios obtained using altered filter efficiencies are added into the same four filter categories. For the two categories comprising new filters, the original simulation has an average *I*/*O* ratio that is closer to the measurement than is the case for the altered simulations. For the categories with aged filters, the results are different. An increased *η* is performing closer to the measurement for the aged filter with ionization category, while a decreased *η* is better for the aged filter no-ionization category. This agrees with the validation results in Fig. 5 that the aged filter ionization group originally underestimates the *η* much, but the new filter groups are already performing well.

**Fig. 10:**
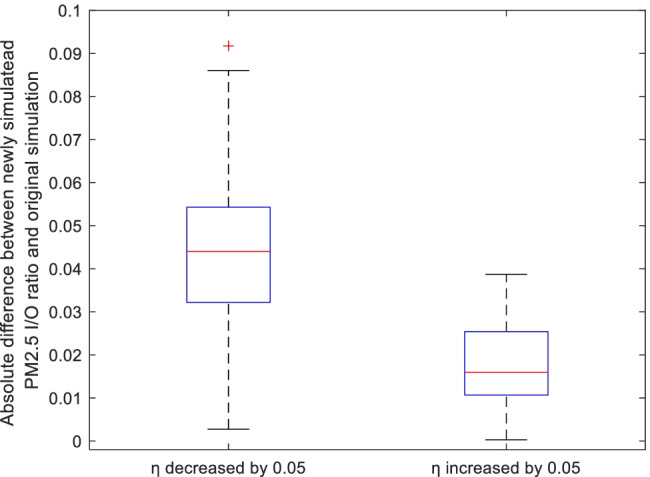
Box-whisker plot of absolute difference between the altered and the original simulations of the average PM_2.5_ *I*/*O* ratios when filter efficiencies were increased and decreased by 0.05. Data include all test cases (128 samples)

Furthermore, all the total of 128 sample cases were compared individually, in the sense of original and altered simulations of the PM_2.5_ *I*/*O* ratio, when the filter efficiency was changed. This is presented as in Fig. 10. When the efficiency was decreased by 0.05, 47 cases reported an absolute change in the PM_2.5_ *I*/*O* ratio larger than 0.05, while all of them smaller are than 0.1. Reversely, when the efficiency was decreased by 0.05, all the cases reported an absolute change in the PM_2.5_ *I*/*O* ratio smaller than 0.05.

It could be concluded that the filter efficiency is a relatively crucial parameter for predicting PM_2.5_ and UFPs in the cabin, especially when the aged filter is simulated.

#### Ventilation airflow

The ventilation from outside air airflow (*Qoa*) and ventilation airflow from recirculation (*Qrec*) in this model are simulated based on the vehicle climate model developed in a previous study (Nielsen et al. 2015), which uses the same control strategies as in the climate control unit of the vehicles tested in the present study. Within the four airflow levels simulated, the Low airflow level is common in normal user setups and contains more available data samples. To investigate the influence from airflow estimation on the model, the airflow rates were varied for all the Low cases in a sensitivity study. *Qoa* and *Qrec* are altered together by ± 10%, ± 30%, ± 50% and ± 70% of original values. This also ensures that the recirculation degree (%), i.e. the relationship between these two, is maintained the same as the original simulation. The results mainly showed that the simulated particle concentration in each size bin (*Cin*), PM_2.5_ and UFPs, as well as *I*/*O* ratios, for all the cases are nearly not changed at all compared to the original simulation results.

To conclude, ventilation airflow variation within a common deviation range in this study is not influencing the simulation to a high degree. One reason is that the influence from ventilation airflow on filter efficiency is not considered in this model due to limited data. Furthermore, when solving Eqs. (1) and (2), *Qoa* exists in both the source and loss terms and is mostly more than 10 times larger than the other airflow items; thus, changing *Qoa* would not directly influence the particle results dramatically. When the high recirculation and infiltration both happen, the particle simulation results would be more sensitive to the *Qrec* variation.

#### Infiltration and deposition

The infiltration airflow (*Qinf*) was estimated from relevant studies using vehicle characteristic values as in Table A2 for the two cars in this study (Lee et al. 2015b). The leakage flow coefficient *kf* and pressure exponent *n* adopted correspond to reported values from similar vehicle types and cabin volumes, which are relatively low. The validation results showed that the infiltration values (*Qinf*) were almost all zero in all 128 data samples, except for a few cases that have *Qinf* in the magnitude of 10^−4^ m^3^/s. This could be expected since, in general, the newer cars are predicted to have better sealing performance and the measurement cases always have the ventilation fan on, which pressurizes the cabin. The cases with positive *Qinf* are all with high recirculation degrees, where cabin pressurization from outside air (*Qoa*) is less.

In our validation data, the ventilation airflows from outside air (*Qoa*) are between 44 and 291 m^3^/h (12–81 L/s) and in 116 of 128 cases they are higher than 58 m^3^/h (16 L/s). The driving speeds are between 13 and 114 km/h in China (S90) with an average of 76 km/h and are all zero in Gothenburg (XC90). Of 128 cases, 120 are below 103 km/h. This supported that our result mainly agrees with studies from Lee et al. (2015a, b), where they concluded that average outside air ventilation airflows between 58 and 133 m^3^/h could prevent infiltration when driving speeds are correspondingly below 103–123 km/h.

**Fig. 11:**
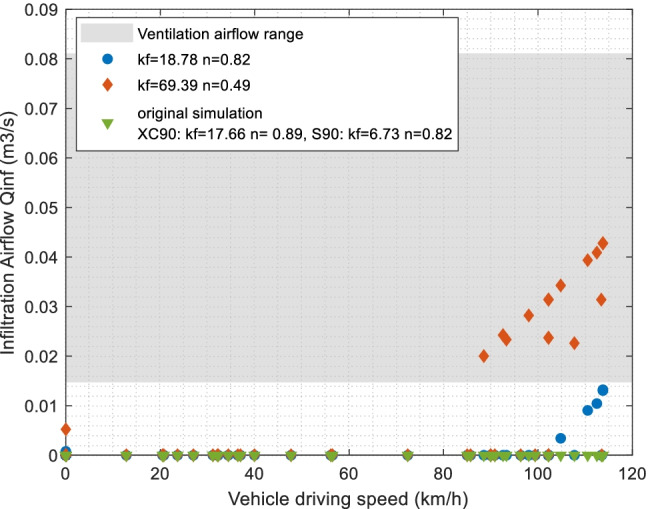
Simulation of infiltration airflow *Qinf* (m^3^/s) vs. vehicle driving speed, when using different *kf* and *n* values, compared with original simulation. At lower speeds of 0–80 km/h, the infiltration airflows are mainly all zero; thus, all the markers are overlapping at *y* = 0 in the figure. The HVAC ventilation airflow (*Qoa* + *Qrec*) range is given as the grey area for comparison. Data include analysis on all test cases (128 samples)

Although the adopted *kf* and *n* values were from similar vehicles and showed expected results, they were still varied in the sensitivity analysis to investigate the variation of *Qinf*. Higher *kf* values from two other vehicle models (Lee et al. 2015b) were utilized, where a *kf* of 69.39 is the maximum in all vehicle models. Figure 11 presents the *Qinf* variations of all test cases using various *kf* values. For comparison, the HVAC ventilation airflow (*Qoa* + *Qrec*) ranges of all test cases are shown, and the range represents four (Xlow to High) ventilation levels that were measured.

When *kf* equals 18.78, the results are similar to those of the original simulation. However, the infiltration is positive for some cases with driving speeds higher than 105 km/h, and these cases all have Xlow or Low fan settings, which mean lower ventilation airflow. When *kf* is 69.39, a few cases above 80 km/h would give *Qinf* values almost equal to the ventilation airflow, and they also have lower fan settings. While this highest *kf* value corresponds to cabin volumes and vehicle types different from our studied vehicles, it could be considered not relevant. To conclude, the simulation of infiltration flow would possibly be more sensitive to high-speed conditions, when *kf* and *n* values are deviating from those relevant for the studied vehicles.

The particle deposition rates *β* (h^−1^) were utilized from relevant studies as in Table A1 (Appendix) in the simulation. The results showed that deposition has a relatively small contribution and the deposition flow *Qdep* was 30–170 times smaller than ventilation airflow (*Qoa* + *Qrec*).

During the sensitivity analysis, the deposition rate *β* (h^−1^) was varied within the literature-reported range of 0.5–12.6 (h^−1^) (Ott et al. 2008; Ding et al. 2016; Harik et al. 2017). A higher *β* (h^−1^) leads to a higher *Qdep*. While using the highest value of 12.6 (h^−1^), *Qdep* is still on average 3–4 times smaller than ventilation airflow for the two studied vehicles.

### Sample modelling for air quality and energy use

Most of today’s passenger cars have a climate system designed to heat up and cool down the cabin in a rather short period of time and then keep the temperature at a desired level. Possible future requirements on in-cabin air quality in passenger cars may require more advanced climate system controls including sensors (e.g. particle and CO_2_ concentrations). Such developments will likely rely on system simulation models that include the parameters that will be involved. The ventilation settings affect the particle and CO_2_ concentrations in the cabin and would also possibly affect the energy use for climate system in the car. For example the air recirculation degree could potentially benefit energy use and reduce particle concentration under certain outdoor conditions, since the HVAC-treated cabin air is reused. Meanwhile, it could also increase CO_2_ levels in the relatively condensed cabin. The following is an initial example of how the developed model can be used to further investigate these relationships under common user-case outdoor conditions.

The studied example case chosen has a measured indoor PM_2.5_ of 48 μg/m^3^ when using the installed aged filter, which was higher than the WHO recommendation of 25 μg/m^3^ 24-h mean.^1^ The measured inside CO_2_ level is 968 ppm with two persons in the cabin. Given the guidelines from ASHRAE (The American Society of Heating, Refrigerating and Air-conditioning Engineers) (ASHRAE 2018) that inside CO_2_ levels should not be more than 700 ppm higher than the outdoor levels, the target value of CO_2_ is set to 1500 ppm for the vehicle cabin, which is also considered the reference in the development of the studied vehicle’s climate strategy.

This case has the setting of aged filter, Low airflow rate and ionization off. The original measured outdoor particle distributions and outdoor temperature are used as model input. The in-cabin desired temperature is 22 °C, and the airflow rate is low (the same as in the measurement), while the recirculation degree was varied, as 0, 30%, 50% and 70%. The corresponding inside PM_2.5_, CO_2_ and steady-state power consumptions are compared. The power consumption refers to the major components in the climate system, i.e. air compressor, blower, air heater and cooling fan.

**Fig. 12:**
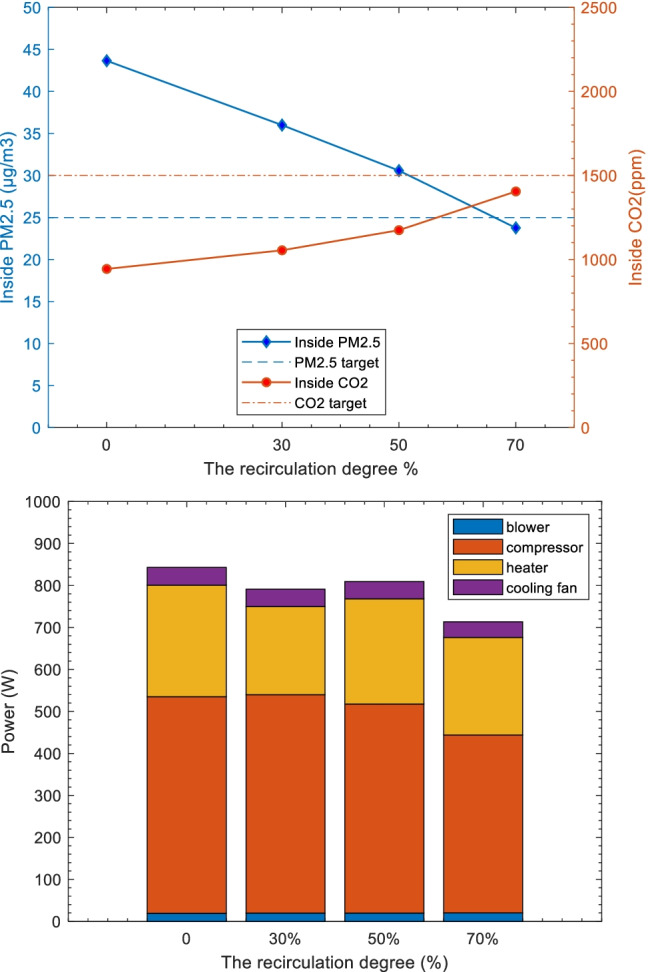
Simulated inside PM_2.5_, CO_2_ concentration and major climate power consumption at varied recirculation degrees (%). The studied example case has these settings: aged filter, Low airflow rate and ionization off

The results are shown in Fig. 12. As recirculation increases, the PM_2.5_ in cabin obviously decreases, and the CO_2_ increases. Considering the CO_2_ target value of lower than 1500 ppm, 70% recirculation reduced the PM_2.5_ level under the target of 25 μg/m^3^ while maintaining an acceptable CO_2_ level. The blower and cooling fan consumptions under these settings are not varying to a high degree. The heater power and compressor power were reduced by around 13% and 18% when recirculation increases from 0 to 70%.

However, the development of advanced ventilation strategies needs further model development, as well as development of suitable driving cycles enabling evaluation of the value of the strategies.

## Discussion

The filter efficiency in real road conditions depends on many factors. Outside particle concentration and distribution (Knibbs et al. 2009), pollutant sources (Kaur et al. 2005; Qiu et al. 2017), filter ageing status and ventilation airflow rate (Abi-Esber and El-Fadel 2013) are among the factors that influence the actual filtration in the vehicle. Thus, using laboratory component data in exclusive standardized test conditions to estimate road testing would originally include possible deviations. Component tests with extended scenarios would be beneficial in better predicting the filter performance. Specifically, regarding this study, the filter efficiency and pressure drop data as a function of ageing status and airflow rate would improve the simulation of particle concentrations and the fan power in the HVAC system.

The 500-h-aged filter was simulated using filter efficiency data available from companion aged filters in different environments for the same model type. The ageing sources of these filters are not the same, since they were all aged with outdoor pollutants instead of standardized dusts. This ageing method is aiming at achieving as close to real road pollutant conditions as possible, but it naturally includes more variance and makes each aged filter not entirely the same. A better controlled ageing environment would be helpful to improve the repeatability, as well as to provide meaningful data for further prediction usage.

The influence from airflow rate or face velocity on filter efficiency was not investigated in this study due to lack of corresponding data. The study from Xu et al. (2011) has reported that change of vehicle fan level from 1 to 5 would decrease the filtration efficiency by 10–20% for particles in the 10- to 50-nm size range. A further simulation considering this could be achieved either from more filter component data, or from estimation based on these similar studies.

The CO_2_ concentration in cabin is simulated based on well-mixed assumption, while it is observed that the mixing requires a longer time than the sampling period of 5–10 min. This would have contributed to the deviation because the measured CO_2_ concentration has not reached the stabilized value. In the future study, the sampling period could be extended. On the other hand, the transient solution of the CO_2_ mass equation can be solved and compared with road measurements for further validation. Besides, it also would be of interest to test with a 100% recirculation degree, to understand the CO_2_ accumulation in this condition and provide inputs for designing the running duration of recirculation cases in the vehicle for example during a quick heat-up period or in tunnel environments.

## Conclusion

In this study, a vehicle cabin air quality model was developed for particles including PM_2.5_ and UFPs, and for CO_2_. Particle mass and count concentrations for particle sizes between 10 nm and 2.5 μm were simulated. The model uses inputs from parameters including outdoor particles/CO_2_ levels, vehicle speed, ventilation airflow (climate settings), filter status, ionization status, passenger numbers etc.

A previously developed model for the same vehicle platform climate system was used to provide inputs of airflows to the air quality model. The filter efficiency was size dependent and varies according to filter age and ionization status. This study also estimates particle deposition and infiltration with experienced vehicle characteristic parameters from corresponding studies.

Previous road tests with the modelled two vehicles were used to validate the model. From the results, it turned out that in general the model simulation correlates well with the measured data with regard to PM_2.5_, UFPs, particle concentration per size channel and CO_2_ even though filter data are incomplete.

Different filter statuses and ionization statuses exist in the validation data. In general, the estimations are good and could reflect the road measurements, except for aged filters with ionization which exhibit relatively higher overestimation of particle concentrations. In the sense of different airflows, the predictions for Medium airflow are better, due to the fact that the filter efficiency values adopted in the model were tested at airflows close to the Medium airflow. When it comes to details in each size channel, model prediction within the particle size range 52–352 nm mostly shows a general overestimation of concentrations in both new and aged filter groups.

The model is used to investigate sample cases further with different recirculation degrees, and the corresponding particle and CO_2_ concentrations are simulated. The climate model simulates the corresponding power consumption of the climate system. This indicates the usefulness of the model to provide inputs for usage of recirculation in the vehicle to improve air quality and improve energy efficiency.

Further studies will be enhanced using improved aged filter efficiency estimations — including the influence from airflow on filter efficiency.

## Supplementary Information

Below is the link to the electronic supplementary material.

Supplementary file1 (DOCX 93 KB)

## Data Availability

The datasets used and/or analysed during the current study are available from the corresponding author on reasonable request.

## Nomenclature

*a*: Coefficient for calculating *dPaero*
*b*: Coefficient for calculating *dPaero*
*Cbr*: Carbon dioxide concentration contained in the passenger’s exhaled air (ppm)
*Cenv*: Outside particle count (N/cm^3^) or mass concentration (μg/m^3^) in one size channel or outside CO_2_ (ppm)
*Cin*: Inside particle count (N/cm^3^) or mass concentration (μg/m^3^) in one size channel or inside CO_2_ (ppm)
*dPaero*: Pressure difference due to aerodynamic characteristics (Pa)
*dPmech*: Pressure difference due to mechanical ventilation (Pa)
*FAC2*: The fraction of predictions within a factor of 2 of observations
*FB*: Fraction bias
*Frev*: Reverse leakage flow correction factor
*kf*: Leakage flow coefficient
*kp*: Aerodynamic pressure distribution coefficient
*MG*: Geometric mean bias
*NMSE*: Normalized mean square error
*N*: Number of passengers in the vehicle
*n*: Pressure exponent
*Qdep*: Deposition flow (m^3^/s)
*Qinf*: Infiltration airflow (m^3^/s)
*Qoa*: Ventilation airflow from outside air (m^3^/s)
*Qps*: Passive ventilation airflow (m^3^/s)
*Qrec*: Ventilation airflow from recirculation (m^3^/s)
*r*: Pearson’s correlation coefficient
*Vbr*: Minute ventilation; the amount of air breathed per minute (L/min)
*Vcabin*: Cabin volume (m^3^)
*VG*: Geometric variance
*α*: Particle penetration loss coefficient
*β*: Particle deposition coefficient (h^−1^)
*η*: Size-dependent filter efficiency
Δ*Pinf*: Pressure difference causing infiltration airflow

## Footnotes

1 This guideline value was updated in September 2021 to be 15 μg/m^3^.
