# Supplementary material for: Size-resolved simulation of particulate matters and CO2 concentration in passenger vehicle cabins
Source: Environ Sci Pollut Res Int. 2022 Feb 10;29(30):45364–79. doi: 10.1007/s11356-022-19078-1 (PMC9209366; doi:10.1007/s11356-022-19078-1)
Supplement: Supplementary file 1 — Supplementary file1 (DOCX 93 KB) [file 11356_2022_19078_MOESM1_ESM.docx]

Appendix

Supplementary data are presented in Appendix.

**Appendix A: Supplementary information of parameter definition in the model formation**

Table A1: Size dependent deposition rates β (h-1) values for particles between 10nm to 2.5 μm

| **Particle Size(μm)** | 0.01^a^ | 0.02 | 0.03 | 0.04 | 0.05 | 0.06 | 0.07 | 0.08 | 0.09 | 0.10 | 0.10-2.5^b^ |
| --- | --- | --- | --- | --- | --- | --- | --- | --- | --- | --- | --- |
| **β (h-1)** | 20 | 16 | 12 | 10 | 8 | 6 | 5 | 5 | 4 | 4 | 0.6 |

^a^ Size-dependant values for particles between 10-100 nm are utilized from relevant UFP study (Xu and Zhu 2009)

^b^ For particles other than UFP, considering the vehicle types, ventilation modes in our measurements, the reported values for ‘Hyundai Verna, AC on fresh air mode’ is used from relevant PM_2.5_ study (Harik et al. 2017)

Table A2: Values of the infiltration estimation parameters for the two simulated vehicles correspondingly

| **Parameter** | **Kf** | **n** | **a** | **b** | **kp** | **Frev** |
| --- | --- | --- | --- | --- | --- | --- |
| XC90 | 17.66^a^ | 0.89 ^a^ | 0.51^c^ | 0.04^c^ | 0.33^d^ | 0.65^e^ |
| S90 | 6.73^b^ | 0.82 ^b^ | 0.51 ^c^ | 0.04 ^c^ | 0.33^d^ | 0.65^e^ |
| Description | leakage flow coefficient | pressure exponent | Coefficient for calculating dPaero | Coefficient for calculating dPaero | aerodynamic pressure distribution coefficient | reverse leakage flow correction factor |

^a^ Based on vehicle types and cabin volumes the reported values of kf and n from ‘Ford Explorer’ is used (Lee et al. 2015a)

^b^ Based on vehicle types and cabin volumes the reported values of kf and n from ‘Nissan Sentra’ is used (Lee et al. 2015a)

^c^ Experiment reported values for sedan type are a=0.54 and b=0.04(Lee et al. 2015a)

^d^ *Frev* was reported as 0.65 for the infiltration flow (Lee et al. 2015a)

^e^ *Kp* was calibrated to be 0.33 for sedan with measured CO_2_ data (Lee et al. 2015a)

**Appendix B: Model performance parameters definition and explanation**

These parameters are used to analyse the performance of the simulation: the Pearson correlation coefficient (r), the fraction of predictions within a factor of two of observations (FAC2), geometric mean bias (MG), the geometric variance (VG), the fractional bias (FB), the normalized mean square error (NMSE).

Pearson correlation coefficient (Pearson’s r) reflects the linear correlation between prediction and observation. It is defined as:

$$\begin{aligned} r= \frac{\sum_{i=1}^{n} \left( Oi- \bar{O} \right)\left( Pi- \bar{P} \right)}{\sqrt{\sum_{i=1}^{n} \left( Oi- \bar{O} \right)^{2}}\sqrt{\sum_{i=1}^{n} \left( Pi- \bar{P} \right)^{2}}}\#\left( 10 \right) \end{aligned}$$

P and O stands for one prediction and observation pairs, and *i* is the index of sample number. $\bar{P}$ and $\bar{O}$ are corresponding average of model prediction and observation.

FAC2 counts the fraction of model predictions that satisfies the criteria:

$$\begin{aligned} 0.5\leq\frac{Pi}{Oi}\leq2 \#\left( 11 \right) \end{aligned}$$

This is a relatively robust measure since it is not overly influenced extreme outliers.

FB and MG are measures of mean bias and indicate the systematic errors. FB reflects the mean bias between prediction and measurement, i.e., an evaluation of overestimation or underestimation. They are defined as:

$$\begin{aligned} FB= \frac{\sum_{i=1}^{n} \left( Oi-Pi \right)}{0.5\sum_{i=1}^{n} \left( Oi+Pi \right)} \#\left( 12 \right) \end{aligned}$$

$$\begin{aligned} MG= e^{\left( \bar{lnO}-\bar{lnP} \right)} \#\left( 13 \right) \end{aligned}$$

NMSE and VG are measures of scatter, i.e., both systematic and random errors. They are defined as:

$$\begin{aligned} NMSE= \frac{\bar{\left( O-P \right)^{2}}}{\left( \bar{OP} \right)} \#\left( 14 \right) \end{aligned}$$

$$\begin{aligned} VG= e^{\left[ \bar{\left( lnO-lnP \right)^{2}} \right]} \#\left( 15 \right) \end{aligned}$$

**Appendix C: Extra results**

**Fig. 13** UFP Indoor to outdoor ratio (I/O ratio) of different parameter groups: the filter statues of new and aged, and the ionization status of on and off are combined. a) Simulated and measured average I/O ratios are presented. b) The absolute difference between simulated and measured I/o ratio of each sample are summarized in box-whisker plots.
